# Immune Checkpoints and Cellular Landscape of the Tumor Microenvironment in Non-Melanoma Skin Cancer (NMSC)

**DOI:** 10.3390/cells13191615

**Published:** 2024-09-26

**Authors:** Ahmed M. Mousa, Alexander H. Enk, Jessica C. Hassel, Robin Reschke

**Affiliations:** 1 Department of Dermatology and National Center for Tumor Diseases (NCT), Medical Faculty Heidelberg, Heidelberg University NCT Heidelberg, a Partnership between DKFZ and University Hospital Heidelberg, 69117 Heidelberg, Germany; 2 German Cancer Consortium (DKTK), DKFZ, Core Center Heidelberg, 69120 Heidelberg, Germany

**Keywords:** immune cells, NMSC, cSCC, BCC, ICI, TME

## Abstract

Non-melanoma skin cancer (NMSC) is primarily categorized into basal cell carcinoma (BCC), the most prevalent form of skin cancer, and cutaneous squamous cell carcinoma (cSCC), the second most common type. Both BCC and cSCC represent a significant health burden, particularly in immunocompromised individuals and the elderly. The immune system plays a pivotal role in the development and progression of NMSC, making it a critical focus for therapeutic interventions. This review highlights key immunological targets in BCC and cSCC, with a focus on immune checkpoint molecules such as PD-1/PD-L1 and CTLA-4, which regulate T cell activity and contribute to immune evasion. This review also highlights anti-tumor immune cell subsets within the tumor microenvironment (TME), such as tumor-infiltrating lymphocytes (TILs) and dendritic cells. Additionally, it examines the immunosuppressive elements of the TME, including regulatory T cells (Tregs), myeloid-derived suppressor cells (MDSCs), tumor-associated macrophages (TAMs), and cancer-associated fibroblasts (CAFs), as well as their roles in NMSC progression and resistance to therapy. Emerging strategies targeting these immune elements, such as monoclonal antibodies, are also discussed for their potential to enhance anti-tumor immune responses and improve clinical outcomes. By elucidating the immunological landscape of BCC and cSCC and drawing comparisons to melanoma, this review highlights the transformative potential of immunotherapy in treating these malignancies.

## 1. Introduction

Skin cancer is broadly classified into two main types: melanoma and non-melanoma skin cancer (NMSC), with NMSC further divided into basal cell carcinoma (BCC) and cutaneous squamous cell carcinoma (cSCC). In recent decades, the incidence of BCC and cSCC has significantly increased across various European regions, particularly in northern and western areas [1,2]. A study using data from the Scottish cancer registry over a 12-year span reported an annual increase of 1.4% to 3.5% in these cancers [3]. Similarly, the Danish cancer registry documented an annual rise of 3.1% to 4.6% over 30 years for BCC and cSCC [4]. In Germany, data from 11 cancer registries indicated annual increases between 3.3% and 11.6% for both BCC and cSCC over 13 years [5]. Research in Germany, the Netherlands, and Scotland found that age-adjusted incidence rates (per 100,000 people annually) for cSCC rose across all populations studied, with annual percentage increases ranging from 2.4% to 5.7%. The most pronounced rise occurred among individuals aged 60 years and older, especially men over 80, where a three-to-five-fold increase was observed [6]. Projections through 2044 suggest a continued rise in incidence rates in all these countries. Age-adjusted mortality rates showed slight increases of 1.4% to 3.2% annually in Saarland and Schleswig-Holstein for both genders, and among men in Scotland. However, in the Netherlands, mortality rates remained stable for women but declined for men [6]. In the United States, the incidence of skin cancer has been steadily rising over the past two decades, with keratinocyte-derived skin cancers accounting for the majority of cases [7]. This trend is expected to continue due to factors such as inadequate sun protection and an aging population [8,9,10]. It is estimated that around 5 million NMSCs are diagnosed annually in the U.S. [11], with BCCs comprising approximately 80% of cases, though they rarely metastasize. Conversely, cSCCs account for about 20% of NMSCs and have an estimated metastasis rate of 2.6% to 9% [12,13]. With over 700,000 new cases annually [10], cSCC is treatable when detected early, but about 4% of patients develop metastases, resulting in a 2% mortality rate, which is comparable to melanoma due to the high initial incidence [14].

Ultraviolet (UV) radiation from the sun is the primary cause of NMSC, with immunosuppression also being a significant factor in the development of aggressive cSCC [15]. Traditional risk factors for cSCC include age, UV exposure, ethnicity, skin phototype, and immunocompromised status [16]. Immunosuppression is closely linked to an elevated risk of developing keratinocyte cancer (KC). Organ transplant recipients (OTRs) are at a particularly high risk, with cSCC rates up to 250 times higher and BCC rates about 10 times higher than in the general population [17,18]. The variation in immunogenicity between these tumors may explain this discrepancy, with cumulative immunosuppressive medication doses having a greater impact on cSCC risk than BCC risk [18,19]. Studies indicate that heart/lung and renal transplant recipients have higher KC rates compared to liver transplant recipients [20,21,22], and the BCC to cSCC ratio in liver transplant patients is closer to that of the general population, likely due to reduced cumulative immunosuppressive doses [20,21,23]. Unlike cSCC, there is insufficient evidence to suggest that BCCs in OTRs exhibit aggressive behavior. Unique features of BCCs in OTRs include earlier onset, more lesions in extra-cephalic locations, and occurrence at unusual sites such as the genitalia and axillae [24]. However, a retrospective study of renal transplant recipients found no differences in the localization or clinicopathologic presentation of BCCs [25].

It is therefore crucial to further investigate the causes and mechanisms underlying NMSC to develop improved strategies for its prevention, detection, and early treatment. In cancer therapy, antibodies targeting immune inhibitory receptors like CTLA-4, PD-1, and PD-L1 have been widely used. Current clinical research is focused on exploring various antibodies and agents targeting immune checkpoint proteins. There is a significant correlation between inhibitory immune checkpoint molecules, i.e., LAG-3 CTLA-4 and PD-Ll, and tumor progression. The surrounding environment of the cancer cells is referred to as the tumor microenvironment, in which tumor cells induce cross-talk with other cell types at cellular and molecular levels [26,27,28,29]. By promoting pro-tumoral immune cell phenotypes that can potentially act as a resistance barrier for immunotherapies, it can result in suppression of the immune response as well as progression of the tumor [30,31]. The tumor microenvironment (TME) includes fibroblasts, dendritic cells, macrophages, and lymphocytes, with the composition varying depending on the cancer type and the patient’s immune status [32]. Each cell type within the TME can exert either pro- or anti-tumoral effects, depending on their interactions with other TME components [28]. This review discusses cSCC-associated dendritic cells, macrophages, myeloid-derived suppressor cells, and T cells, alongside immune checkpoint molecules involved in modulating T cells within the TME and their role in immune evasion and potential for therapeutic blockade.

## 2. Key Immune Cells in the Tumor Microenvironment of NMSC

### 2.1. Dendritic Cells

Dendritic cells (DCs) play a significant role in connecting the innate and adaptive immune systems, thus are crucial for cancer immune surveillance [31,33]. In humans, three cutaneous DC subsets exist: Langerhans cells (LCs), myeloid DCs (mDCs), and plasmacytoid DCs (pDCs) [34]. LCs, found in the epidermis, are the initial antigen-presenting cells (APCs) to encounter cSCC [35]. Evidence suggests that despite environmental influences, the ability of LCs to induce a robust immune response may be sustained [36]. In contrast, dysfunctional dermal DCs (dDCs) play a potential role in the failure to initiate adequate cytotoxic responses [36]. Additionally, tumor-induced strategies such as DC dysfunction and apoptosis are employed by tumors to evade immune surveillance [37,38,39,40]. Specifically, cSCC lesions exhibit reduced numbers of LCs and CD11c^+^ dDCs [41,42], along with the impaired ability of dDCs to activate T cells and stimulate interferon (IFN)-g production [41,43]. Immunoregulatory cytokines, such as TGF-b, IL-10, IL-6, and VEGF-A, are thought to contribute to mDC suppression [41]. Moreover, the SCC microenvironment is characterized by a high presence of plasmacytoid DCs (pDCs) [41], which produce significant amounts of IFN- α in response to foreign antigens and may play a crucial role in anti-tumor immune responses [44,45,46]. Overall, the pivotal role of DCs in connecting innate and adaptive immunity, along with their ability to initiate immune responses, presents them as promising targets for tumor immunotherapy [31,35]. Particularly interesting is that they produce relevant amounts of chemokines such as CXCL9 and CXCL10, which can recruit circulatory effector T cells to the TME by binding to their cognate receptor CXCR3 [47].

### 2.2. Tumor-Infiltrating T Cells (TILs)

Tumor-infiltrating T cells (TILs) are prognostically relevant biomarkers for ICI therapy across tumors [48]. NMSC (cSCC and BCC) showed significantly more Th1 and Th17 cells than normal skin [49]. In particular, IFN-γ-producing CD8+T cells and depletion of γδ T cells were observed. NMSCs featured denser T cell infiltrates (CD4+, CD8+, and Tregs) than normal skin [49]. The peritumoral region of cSCC and transplant-associated SCC (TSCC) exhibits a significantly higher density of CD3+ and CD8+ T cells compared to normal skin, while the tumor region itself contains a lower number of these cells [50]. The Th1-dominated immune response in NMSC argues for deploying immunotherapies such as ICI in advanced NMSC. It is conceivable that a peritumoral infiltration with T cells can be sufficient for tumor cell killing induced by ICI with a response rate of up to 50% in cSCC [51].

### 2.3. Tissue-Resident Memory T Cells (TRMs) 

In a recent study, it was demonstrated that CD8^+^ tissue-resident memory T cells (TRMs) in cSCC have the capacity to produce IFNγ, TNFα, and IL-2, suggesting potential immunostimulatory functions [52]. However, these cells also exhibited an enhanced production of immunosuppressive cytokines such as IL-10, the ectonucleotidase CD39, and upregulation of the exhaustion marker PD-1 [53]. Similarly, these TRMs showed elevated expression of other relevant inhibitory checkpoint molecules such as CTLA4, LAG-3, and Tim-3, suggesting a potential role for checkpoint inhibition and re-invigoration of this T cell subset [52,54]. Importantly, their association with poorer clinical outcomes in cSCC calls for a comprehensive understanding of their function in the TME [53]. These findings contrast with studies on other types of cancer such as melanoma, where CD8^+^ TRMs have been shown to be critical for early protection and response to ICI [55]. Elevated levels of CD8^+^CD103^+^ cells have been observed to be linked with metastasis, and it has been noted that CD8^+^CD103^+^ TRMs are more prevalent in cSCC compared to non-lesional skin [53]. Further research needs to clarify the role of TRMs in ICI-treated metastasized or locally advanced NMSC (cSCC and BCC).

### 2.4. Regulatory T Cells (Tregs)

**Figure 1:**
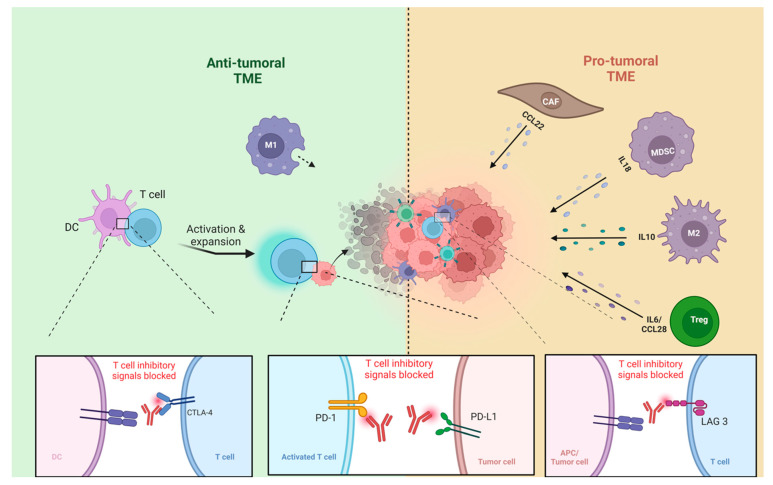
TME of NMSC: pro-tumoral TME vs. anti-tumoral TME, tumor-associated macrophages type1 (M1), tumor-associated macrophages type 2 (M2), dendritic cells (DC), myeloid-derived suppressor cells (MDSCs), regulatory T cells (Tregs), antigen-presenting cell (APC). Figure created with BioRender.com.

Compared to normal skin, which has a lower population of FOXP3+ Treg cells, TSCC and cSCC exhibit an increased presence of these cells [56]. FOXP3+ Treg cells play a critical role in maintaining immune homeostasis [57], preventing autoimmune diseases [58]. However, they also contribute to a pro-tumor environment by suppressing anti-tumor immunity [59,60]. Their presence in the TME is likely due to their recruitment from the bloodstream, as they do not proliferate locally within the tumor [61]. In immunocompromised patients, FOXP3+ Treg cells constitute more than half of the T cell infiltrates in cSCCs. These cells express markers such as CCR7 and L-selectin [50] (Figure 1). Specifically, Treg cells can regulate the immune response by suppressing the proliferation and cytokine production of effector T cells [62,63]. Several studies have indicated that a higher number of tumor-infiltrating Tregs is associated with poor prognosis and lower survival rates in breast, ovarian, and gastric carcinomas [59,64,65,66]. Tregs may play a role in cSCC metastasis and potentially have prognostic significance [67]. In cSCC, the regulatory activity of CD8+ Tregs is more pronounced compared to that of CD4+ Treg cells [68,69,70]. Imiquimod has been found to effectively inhibit the immunological destruction of cSCC by decreasing the number of FOXP3+ Treg cells and inhibiting Treg cell function [61]. Research findings indicate that BCC samples exhibit a high presence of tumor-associated Treg cells in the TME while having a relatively low proportion of stromal tumor-infiltrating lymphocytes (sTILs) [71] This is supported by another study in which BCCs showed a high stromal FoxP3+ to CD4+ T cell proportion [72]. Pro-tumoral TMEs are characterized by a dominance of Treg cells and other immunosuppressives cells (Table 1) [73]. Whereas, anti-tumoral TMEs are populated by more CD8+ T cells and dendritic cells, among others (Figure 1) [73]. These TME classifications have implications for immunotherapy response, with anti-tumoral TMEs generally showing better outcomes in response to ICI than pro-tumoral TMEs [74]. Therefore, alternative therapeutic strategies for pro-tumoral TMEs need to be further explored [71,75].

### 2.5. Myeloid-Derived Suppressor Cells (MDSCs)

The development of myeloid-derived suppressor cells (MDSCs) is driven by signals that occur in two distinct but partially overlapping phases [82]. The first phase involves the expansion of immature myeloid cells, while the second phase sees the conversion of neutrophils and monocytes into pathologically activated MDSCs [82]. In the context of cancer, MDSCs play a significant role in immune suppression, leading to tumor progression and resistance to immunotherapy [74]. Factors such as arginase, nitric oxide (NO), and reactive oxygen species (ROS) have been implicated in the T cell suppression mediated by MDSCs [83]. Specifically, MDSCs are critical sources of NO in cSCC, which suppresses E-selectin expression on tumor vessels, thereby limiting the infiltration of skin-homing T cells into tumors and allowing cSCC to evade immune detection [84]. High-risk SCC were associated with increased numbers of both circulating and tumor-resident populations of neutrophils and/or G-MDSC [85]. Successful cancer immunotherapy hinges on the effective elimination of immune suppressive factors from the body, with MDSCs representing a major target [74]. The challenge is developing strategies that can efficiently and selectively target MDSCs in cancer immunotherapy.

### 2.6. Tumor-Associated Macrophages (TAMs)

Macrophages play a crucial role in infiltrating tumor cells, influencing various carcinogenesis stages such as initiation, growth, invasion, and metastasis [86,87,88]. In SCC, an increased abundance of macrophages is observed compared to normal skin [89,90]. These macrophages, referred to as tumor-associated macrophages (TAMs), are found in the vicinity of the tumor and within its microenvironment. The population of macrophages includes two distinct forms: M1, which exhibits anti-tumorigenic properties, and M2, which displays pro-tumorigenic characteristics. Tumor metastasis and progression are linked to the M2 subtype of macrophages, known as tumor-associated macrophages (TAMs). These cells also activate tumor-promoting genes in BCC lesions, further supporting cancer development [91]. On the other hand, M1-like macrophages act as an anti-tumoral cells and induce protection against infections [92]. Notably, investigations into the BCC microenvironment have revealed a prevalence of M2 macrophages over other macrophage types, indicating an M2-dominant, tumor-promoting TME [93]. Similarly, in cSCC tumors, M2 macrophages predominate, fostering an oncogenic milieu [94]. These macrophages exhibit reduced antigen-processing capabilities compared to M1 macrophages, resulting in less effective stimulation of an anti-tumorigenic immune response in cSCC [94]. Furthermore, M2 macrophages play a role in promoting tumor growth in the cSCC TME by stimulating angiogenesis and tissue remodeling [89].

### 2.7. Cancer-Associated Fibroblasts (CAFs)

The fibroblasts within the TME of BCC are termed cancer-associated fibroblasts (CAFs) and are activated and transformed due to prolonged exposure to UV radiation and signals from the tumor [95,96]. These CAFs produce various signaling molecules and proteins that suppress the body’s anti-tumor response [96,97,98]. For instance, they release CXCL12 and CCL22 to hinder the infiltration of CD4^+^ and CD8^+^ T cells while attracting regulatory T cells (Tregs) instead [96]. Additionally, CAFs exhibit a diverse range of phenotypes, displaying plasticity within the TME [99]. Studies comparing CAFs from human SCC tumors with normal human skin fibroblasts revealed an inflammatory gene expression signature unique to cSCC-derived CAFs [100]. Furthermore, research has demonstrated the capability of CAFs to initiate and perpetuate inflammation, heighten cSCC’s invasiveness, and facilitate the formation of new blood vessels (angiogenesis) [76,101].

## 3. Checkpoint Molecules

### 3.1. Programmed Death-Ligand 1 (PD-L1) 

The expression of PD-L1 in sun-exposed regions of cSCC patients is significantly higher compared to non-exposed regions [102]. This finding supports the use of PD-1 and PD-L1 inhibitors as immunotherapy for cSCC. Notably, PD-L1 expression is most prevalent in metastatic cSCC cases [102,103,104,105]. Research indicates that PD-L1 protein expression in cSCC ranges from 20% to 70% of tumors, depending on the tumor’s grade [102,103,104,105]. In these studies, PD-L1 positivity in tumor cells is defined as staining of ≥1% [103]. This immunotherapy approach demonstrated significant efficacy in specific cohorts of cancer patients, such as those with breast cancer, bladder cancer, lung cancer, and melanoma, but also including locally advanced and metastasized NMSC [106,107,108]. Reactions to immunotherapy are frequently encountered among patients, with common adverse events including immune-related (ir) dermatitis or ir-colitis, which can be mediated by TRM cells [81]. It has been observed that new cSCC lesions can occur during immunotherapy targeting PD-1/PD-L1, even though such therapies have successfully improved survival and reduced metastasis [109,110,111,112,113]. Despite these outcomes, research into the potential of ICIs as neoadjuvant or adjuvant therapies for cSCC continues and holds great potential in treating locally advanced or metastasized epithelial tumors such as cSCC. Furthermore, clinical studies are exploring the effectiveness of ICIs in combination with radiotherapy, EGFR inhibitors, or PARP inhibitors for the treatment of cSCC [114]. In several studies, PD-L1 expression in BCCs has been observed to vary significantly, with some reporting high expression levels and others reporting undetectable levels. Chang et al. found that 89.9% of BCC tumors exhibited PD-L1 staining using a cutoff of ≥5% [115]. They also noted that treatment-naïve tumors showed lower staining intensity than those previously treated [116]. Conversely, a recent study found that 42% of cSCC samples exhibited positive staining for membranous PD-L1. In contrast, the researchers observed no membranous PD-L1 staining in BCC samples [117]. These discrepancies could be due to variations in the antibodies used for staining, differences in staining conditions, or differences in criteria for scoring positivity. Managing metastasized or advanced BCC can be challenging and often results in treatment resistance and adverse effects from long-term therapies. For example, hedgehog inhibitors (HHIs) like vismodegib and sonidegib, despite their efficacy in treating advanced BCC, can cause side effects such as muscle spasms, hair loss, taste changes, and fatigue [108,118,119]. In contrast, early-stage BCC can often be effectively treated with minimal intervention. Patients with superficial lesions may benefit from topical immune stimulants, such as imiquimod [120]. Emerging as crucial second-line therapeutic options for patients unresponsive to HHIs and necessitating alternative interventions, anti-PD1 blockade has garnered significant attention [108]. Cemiplimab stands as the lone ICI approved for treating patients with HHI-refractory BCC. A phase II multi-center, open-label trial was conducted, which led to a 31% response rate on advanced non-resectable BCC patients; furthermore, 6 out of 84 patients exhibited complete tumor clearance [108]. Notably, PD-L1 tumor expression did not seem to influence cemiplimab’s efficacy, and tumor regression response to treatment could be considerably delayed [108]. Similarly, patients with metastatic BCC demonstrated a response to cemiplimab, albeit with a lower overall response rate of 21.4%, with no instances of complete tumor clearance [108]. For patients with metastatic or locally advanced BCC who are either unresponsive to HHIs or cannot tolerate them due to toxicity, cemiplimab has been approved as an alternative intervention [115].

### 3.2. Cytotoxic T-Lymphocyte-Associated Protein 4 (CTLA-4)

CTLA-4, a suppressive immune checkpoint receptor primarily located on T cells, is upregulated after T cell activation in order to dampen T cell activity [121]. CTLA-4, also known as CD152, is a co-receptor on T cells [122]. It has a higher binding affinity for B7 family molecules, such as CD80 and CD86, on antigen-presenting cells (APCs) compared to CD28 [122]. The surface of regulatory T cells (Tregs) is a primary site for the expression of CTLA-4 [121,123,124], which plays a crucial role in their development and regulation [123]. CTLA-4 inhibitors function by reducing the cell-mediated immunosuppression exerted by Tregs and enhancing the activity of effector CD4+ T cells [123]. Typically, CTLA-4 activation requires a costimulatory signal when its antigen binds to a naïve T cell, but it is predominantly activated through TCR stimulation [121]. The absence of CTLA-4 has been associated with abnormal T cell proliferation [122]. Additionally, research has explored the potential of CTLA-4 inhibitors to enhance immune responses against tumor cells [125,126,127]. A study found that patients with a genetic variant in the CTLA-4 gene (long-repeat (AT)n variants) were more protected against BCC and cSCC [128]. Combinatorial checkpoint blockade could be explored for therapy refractory NMSC. It was postulated that the concurrent blocking of CTLA-4, which primarily regulates T cell activation and suppresses DC activity via Treg cells, along with PD-1 blockade, which is mainly involved in inhibiting effector T cell and NK cell activation and inducing Treg cell differentiation, would work synergistically [129,130,131,132,133].

### 3.3. Lymphocyte Activation Gene-3 (LAG-3)

LAG-3 represents another important inhibitory checkpoint molecule that can potentially be blocked [134]. Like PD-1 and CTLA-4, LAG-3 regulates T cell activity, dampening immune responses and enabling tumors to evade detection [134]. Higher LAG-3 expression in melanoma correlated with longer progression-free survival (PFS) compared to tumors with less than 1% LAG-3 expression [135,136]. Co-expression of LAG3 with PD-1 is found in exhausted CD8+ T cells, which exhibit reduced IFN-gamma production, suggesting potential therapeutic synergy between PD-1 and LAG-3 inhibitors [136]. In locally advanced BCC, longitudinal biopsies taken during the course of treatment revealed a progressive rise in LAG-3 expression following anti-PD-1 therapy [136]. This pattern supports the concept of targeting LAG-3 alongside PD-1, which could enhance the therapeutic effects of anti-PD-(L)1 treatment in therapy refractory NMSC.

## 4. Distinctive Characteristics of the TME in NMSC and Melanoma

### 4.1. Neutrophils

Melanoma patients and mouse models treated with ICI showed profound neutrophil activation, which can be evidence to support that the complete elimination of tumors relies on neutrophils and, to some extent, on inducible nitric oxide synthase [79]. Analyses using flow cytometry and transcriptomics uncovered a specific type of neutrophil subset that has an anti-tumor activity in treated mice. These results reveal an interaction between T cells and neutrophils, which diminish variants of tumor antigen loss [79]. Another study translated preclinical results to a group of patients with melanoma. This highlights the Ly6Ehi neutrophils’ ability to prognosticate human response to ICI accurately [80]. In a melanoma mouse model, β-glucan was shown to reprogram neutrophils into an anti-tumor phenotype, and this process relied on the bone marrow neutrophil precursor memory [137,138]. On the other hand, neutrophils were found to induce the growth and development of cancer cells in human samples and animal models such as zebrafish by releasing PGE2, which acts as a nutrient factor [137,139]. In addition, neutrophils also demonstrated pro-tumor activity through neutrophil extracellular traps (NETs), which were produced by neutrophils, accumulated in the tumor, and were associated with increasing tumor size in melanoma [140]. The role of neutrophils in NMSCs treated with ICI needs to be elucidated.

### 4.2. Tissue-Resident Memory T Cells (TRMs)

In a recent study, It was found that CD39+ TRM cells were positioned in much closer proximity to melanoma cells than bystander T cells [141]. Patients who had a high percentage of CD39+ TRM cells within the tumor exhibited significantly improved rates of recurrence-free survival compared to individuals with lower proportions [142]. Another study also showed that patients’ sentinel lymph node metastases displayed TRM cell signatures, which were positively associated with survival [143]. In the context of metastatic disease, TRM cells are a crucial target for ICI due to their high expression of inhibitory checkpoint molecules. Following ICI treatment, melanoma patients experience reactivation and expansion of TRM cells within the tumor [81]. In the beginning of anti-tumor immunity, TRM cells help maintain immune balance and avoid the development of primary melanomas [81]. A mouse model revealed the presence of TRM cell formations in skin-draining lymph nodes, which defended against melanoma tumor seeding in the lymph nodes [143]. Furthermore, mice lacking TRM cells were more prone to the development of melanoma [55]. These findings contrast with the elevated levels of TRM cells observed in progressive cSCC [58].

### 4.3. Eosinophils

Eosinophils have various mechanisms of action for anti-tumor activity, and in some cases, they also act as pro-tumor agents. Their anti-tumor activity can be mediated by the release of chemokines [142], which recruit CD8+ T cells in addition to their cytotoxic granular constituents [142,144]. Eosinophils also stabilize the TME by differentiation of the macrophages from the M2 to M1 type, which reduces tumor angiogenesis [142]. Those mechanisms work together to improve survival in melanoma murine models [77,142]. However, the pro-tumor activity is linked to eosinophils’ overproduction of the extracellular traps [77].

### 4.4. Tumor Cells

In addition to examining immune cells as prognostic indicators, tumor-cell-related proteins can also play a crucial role. Studies have identified several signature biomarkers in malignant melanoma, including the absence of β-Catenin and MTAP, along with the presence of Cox-2, Bcl-X, PTEN, Bax, and CD20+ as significant markers [145,146]. These biomarkers have been shown to predict patient survival in malignant melanoma. Furthermore, biomarkers like Bcl-x, COX-2, and MTAP have demonstrated direct therapeutic significance [145,146]. While melanoma is associated with these seven tumor cell protein signatures, equivalent prognostic biomarkers are still lacking for non-melanoma skin cancers (NMSC).

## 5. Discussion

The immune system plays a crucial yet intricate role in the pathogenesis and progression of cSCC and BCC. Research involving both immunocompetent and immunosuppressed individuals has been instrumental in unraveling the immune mechanisms underlying cSCC [19,23]. The clinical visibility of premalignant lesions offers a unique opportunity for early diagnosis and a deeper understanding of the initial events that precede cSCC development. Comprehensive investigations of both premalignant and malignant tissues have the potential to significantly enhance our understanding of the proteomic, genomic, and immunological landscapes of cSCC and BCC. Recent studies have increasingly focused on the TME in cSCC and BCC, revealing the complex interactions between tumor cells and host immune cells. This growing body of knowledge has led to significant advancements in immunotherapy for metastatic cSCC and BCC, including the development of drugs like cemiplimab, which targets the PD-1/PD-L1 pathway. In certain tumors, the TME of NMSC may be rich in immunosuppressive cells such as Tregs, M2-oriented macrophages, and MDSCs, which suppress immune responses and promote tumor invasion and metastasis. In these cases, combination immunotherapy, such as combining anti-PD-1 therapy with anti-CTLA-4 or anti-LAG3 agents, may offer a strategy to overcome the immunosuppressive environment in non-responsive tumors. This approach is supported by findings of increased LAG3 expression in anti-PD-1-refractory locally advanced BCC [147]. Emerging techniques with spatial resolution of the TME are revolutionizing cancer immunology, enabling the discovery of new immunotherapy targets. However, further research is required to develop strategies for targeting pro-tumoral TMEs and overcoming primary resistance to anti-PD-1 treatments. The use of additional immune checkpoint inhibitors (ICIs), such as anti-CTLA-4 and anti-LAG3, may offer benefits, though it is crucial to discuss potential immune-related adverse events with patients beforehand. Notably, the combination of anti-LAG3 and anti-PD-1 may be more advantageous, as it has shown a better safety profile in advanced melanoma compared to the combination of anti-CTLA-4 and anti-PD-1 [134]. In melanoma and other solid tumors, emerging immunotherapy approaches are utilizing bispecific molecules and targeting cytokines and chemokines to effectively address “cold” tumors [148,149,150]. While the efficacy of new treatments is promising, ensuring safety remains a critical priority. Larger trials and further translational research are needed to thoroughly investigate the effect and safety of novel immunotherapies on immune and tumor cells in NMSC.

## 6. Conclusions

The article highlights the crucial role of key immune cells within the TME of NMSC, identifying both pro- and anti-tumor functions. The importance of immune checkpoints is emphasized, along with current therapeutic approaches involving ICI. However, further research is needed to uncover novel therapeutic strategies and TME-based targets for NMSC.

## Figures and Tables

**Table 1:** Overview of the role of key immune cells within the tumor microenvironment of different skin cancers.

Cell Type	Function	Potential Effect on the Tumor		
		cSCC	BCC	Melanoma
**Tregs** [50]	Create an immunosuppressive environment. T cells Suppression	Pro-tumoral	Pro-tumoral	Pro-tumoral
**TAMs** [50,76]	Tumor growth and metastasis (M2 phenotype)	Pro-tumoral	Pro-tumoral	Pro-tumoral
**CAFs** [76]	Secrete factors that downregulate anti-tumor response, recruit regulatory T cells.	Pro-tumoral	Pro-tumoral	Pro-tumoral
**MDSCs** [36]	Promote cancer cell invasion, metastasis, and immunosuppression	Pro-tumoral	/	Pro-tumoral
**Eosinophils** [77,78]	Recruitment of T cells, extracellular traps	/	/	Anti-/or pro-tumoral
**Neutrophils** [79,80]	Inducible nitric oxide synthase, NETs	/	/	Anti-/or pro-tumoral
**TRMs** [81]	Immune equilibrium	Pro-tumoral	/	Anti-tumoral

Figure legend: tumor-associated macrophages type 2 (M2), dendritic cells (DC), myeloid-derived suppressor cells (MDSCs), Regulatory T cells (Tregs), Cancer-associated fibroblast (CAF), TRMs = Tissue-resident memory T cells, / = no data.
